# Supplementary material for: Crash landing of Vibrio cholerae by MSHA pili-assisted braking and anchoring in a viscoelastic environment
Source: eLife. 2021 Jul 2;10:e60655. doi: 10.7554/eLife.60655 (PMC8282333; doi:10.7554/eLife.60655)
Supplement: Supplementary file 1. [file elife-60655-supp1.docx]

Supplementary information for

**Crash landing of *Vibrio cholerae* by MSHA pili-assisted braking and anchoring in a viscoelastic environment**

Wenchao Zhang^1,#^, Mei Luo^2,#^, Chunying Feng^1^, Huaqing Liu^1^, Hong Zhang^1^, Rachel R. Bennett^3,*^, Andrew S. Utada^4,5,*^, Zhi Liu^2,*^, Kun Zhao^1,*^

*^1^ Frontier Science Center for Synthetic Biology and Key Laboratory of Systems Bioengineering (Ministry of Education), School of Chemical Engineering and Technology, Tianjin University, Tianjin, P.R. China*

*^2^ Department of Biotechnology, College of Life Science and Technology, Huazhong University of Science and Technology, Wuhan, China*

*^3^ School of Mathematics, University of Bristol, Bristol, UK*

*^4^ Faculty of Life and Environmental Sciences, University of Tsukuba, Ibaraki , Japan*

^*^ Address correspondence to: rachel.bennett@bristol.ac.uk, utada.andrew.gm@u.tsukuba.ac.jp, zhiliu@hust.edu.cn, or kunzhao@tju.edu.cn.

^#^ These authors contributed equally.

**This file includes:**

Tables S1

**Table S1. Plasmids and primers used in this study.**

| **Plasmids** | **Description** | **Source or reference** | |
| --- | --- | --- | --- |
| pWM91  pML3 | Suicide vector  pWM-FlaAA106C | | *(Metcalf et al., 1996)*  This study |
| pML4 | pWM-FlaAS107C | | This study |
| pML5 | pWM-FlaAA106CS107C | | This study |
| **Primer Name** | **Primer Sequence (5'→ 3')** | | **Description** |
| VC0409-F1 | CTTGTATGGCGCACTCAACG | | *mshA* knockout |
| VC0409-R1-3S | CAGCGCTAATTCAGTTTAAGCGGCCATAGCTACGCAGCATTACTGCAAGG | | *mshA* knockout |
| VC0409-F2-3S | GCTATGGCCGCTTAAACTGAATTAGCGCTGCGTTATACAGCTGCAACCTC | | *mshA* knockout |
| VC0409-R2 | CAAGCATAGCCTTGCTGTTC | | *mshA* knockout |
| VC0409-Mut-T70C-R1 | GTCTAAACATTCAATGCCTTTAATTGCAGCTCGTCC | | MshAT70C construction |
| VC0409-Mut-T70C-F2 | GGCATTGAATGTTTAGACTACACAGCATATAC | | MshAT70C construction |
| VC0409-Mut-Seq-F1 | GGCGAAGAAAGCCAGTATTG | | MshAT70C detection |
| VC0409-Mut-Seq-R1 | CCTGCGGAGAAACTTGAATG | | MshAT70C detection |
| VC2188-F1 | CCATGAGACGGTTCGTTTAC | | *flaA* knockout |
| VC2188-R1-3S | CAGCGCTAATTCAGTTTAAGCGGCCATAGCGATAACGTTGTGCGGTCATC | | *flaA* knockout |
| VC2188-F2-3S | GCTATGGCCGCTTAAACTGAATTAGCGCTGCAGTAGTTCACGGTACCTTC | | *flaA* knockout |
| VC2188-R2 | CCAAAGATGCCGGTAAATGG | | *flaA* knockout |
| VC2188-Mut-F1 | CACACTTTGGTTTCCGGTAC | | FlaA mutations construction |
| VC2188-Mut-R2 | TCCGCACCATTATTGAGAGC | | FlaA mutations construction |
| VC2188-Mut-A106C-R1 | TGACGCTCTGAACATGAGTTGGTACCGTTCGCCGA | | FlaAA106C construction |
| VC2188-Mut-A106C-F2 | AACGGTACCAACTCATGTTCAGAGCGTCAGGCTC | | FlaAA106C construction |
| VC2188-Mut-S107C-R1 | TGACGCTCACACGCTGAGTTGGTACCGTTCGCCGAT | | FlaAS107C construction |
| VC2188-Mut-S107C-F2 | AACGGTACCAACTCAGCGTGTGAGCGTCAGGCTCTG | | FlaAS107C construction |
| VC2188-Mut-A106C-S107C-R1 | TGACGCTCACAACATGAGTTGGTACCGTTCGCCGAT | | FlaAA106CS107C construction |
| VC2188-Mut-A106C-S107C-F2 | AACGGTACCAACTCATGTTGTGAGCGTCAGGCTCTG | | FlaAA106CS107C construction |
| VC2188-Mut-E332C-R1 | CGACGCACACACGTTCTCCTGAATATTCGACAG | | FlaAE332C construction |
| VC2188-Mut-E332C-F2 | ATATTCAGGAGAACGTGTGTGCGTCGAAAAGTC | | FlaAE332C construction |
| VC2188-Mut-G23C-R1 | GTTAAGCTCACACGTCGCCTTGGTCAGATAACGTTGTG | | FlaAG23C construction |
| VC2188-Mut-G23C-F2 | TATCTGACCAAGGCGACGTGTGAGCTTAACACCTCCA | | FlaAG23C construction |
| VC2188-Mut-N26C-R1 | TCCATGGAGGTACAAAGCTCTCCCGTCGCCTTGGT | | FlaAN26C construction |
| VC2188-Mut-N26C-F2 | ACGGGAGAGCTTTGTACCTCCATGGAACGCCTCTCA | | FlaAN26C construction |
| VC2188-Mut-N83C-R1 | GTCGATTCACACATCGCACCTTCTGCGGTTTGAG | | FlaAN83C construction |
| VC2188-Mut-N83C-F2 | AGAAGGTGCGATGTGTGAATCGACCAGCATTTTGCAGC | | FlaAN83C construction |
| VC2188-Mut-S325C-R1 | GTTCTCCTGAATATTACACAGGTTACTGATGCTGTGAC | | FlaAS325C construction |
| VC2188-Mut-S325C-F2 | ATCAGTAACCTGTGTAATATTCAGGAGAACGTGGAAGCGTC | | FlaAS325C construction |
| VC2188-Mut-S87C-R1 | CGCTGCAAAATACAGGTCGATTCATTCATCGCACCT | | FlaAS87C construction |
| VC2188-Mut-S87C-F2 | GAATCGACCTGTATTTTGCAGCGTATGCGTGACCTC | | FlaAS87C construction |
| VC2188-Mut-S376C-R1 | GTGAACTACTGCAATAAACAGATTGCAGAGTTTGGC | | FlaAS376C construction |
| VC2188-Mut-S376C-F2 | TGCAATCTGTTTATTGCAGTAGTTCACGGTACCTTC | | FlaAS376C construction |
| VC2188-Mut-V117C-R1 | ATCTTGCAGTGCACACGACTCTTCATTCAGAGCCTG | | FlaAV117C construction |
| VC2188-Mut-V117C-F2 | GAAGAGTCGTGTGCACTGCAAGATGAACTGAACCGTA | | FlaAV117C construction |
| VC2188-Mut-Seq-F1 | TGAGCTTGCGAACTCGATAG | | FlaA mutations detection |
| VC2188-Mut-Seq-R1 | CGTTCTTCAGCGGATGATAG | | FlaA mutations detection |
